# Transcriptional robustness and protein interactions are associated in yeast

**DOI:** 10.1186/1752-0509-5-62

**Published:** 2011-05-05

**Authors:** Michaël Bekaert, Gavin C Conant

**Affiliations:** 1 Division of Animal Sciences, University of Missouri, 920 East Campus Drive, Columbia, MO 65211, USA; 2 Informatics Institute, University of Missouri, 920 East Campus Drive, Columbia, MO 65211, USA

**Keywords:** Aneuploidy, Dosage balance, Epistasis, Protein-Protein Interactions, *Saccharomyces cerevisiae*

## Abstract

**Background:**

Robustness to insults, both external and internal, is a characteristic feature of life. One level of biological organization for which noise and robustness have been extensively studied is gene expression. Cells have a variety of mechanisms for buffering noise in gene expression, but it is not completely clear what rules govern whether or not a given gene uses such tools to maintain appropriate expression.

**Results:**

Here, we show a general association between the degree to which yeast cells have evolved mechanisms to buffer changes in gene expression and whether they possess protein-protein interactions. We argue that this effect bears an affinity to epistasis, because yeast appears to have evolved regulatory mechanisms such that distant changes in gene copy number for a protein-protein interaction partner gene can alter a gene's expression. This association is not unexpected given recent work linking epistasis and the deleterious effects of changes in gene dosage (*i.e.*, the dosage balance hypothesis). Using gene expression data from artificial aneuploid strains of bakers' yeast, we found that genes coding for proteins that physically interact with other proteins show less expression variation in response to aneuploidy than do other genes. This effect is even more pronounced for genes whose products interact with proteins encoded on aneuploid chromosomes. We further found that genes targeted by transcription factors encoded on aneuploid chromosomes were more likely to change in expression after aneuploidy.

**Conclusions:**

We suggest that these observations can be best understood as resulting from the higher fitness cost of misexpression in epistatic genes and a commensurate greater regulatory control of them.

## Background

Robustness is a common and characteristic feature of living systems [1,2]. It has been extensively studied in the context of gene expression [1], where cells are able to maintain appropriate expression in the presence of both internal and external noise perturbing the transcriptional regulatory machinery [3]. It appears that cells have evolved a number of transcriptional mechanisms to imbue gene expression with robust properties. For instance, negative autoregulation can dampen the effects of network perturbations [4,5]. Similarly, the transcriptional regulatory network contains abundant small circuit motifs (sets of three to five genes with common patterns of regulatory interactions, such as feed-forward loops) [6,7] that have noise-reducing properties [8-10]. Finally, large-scale network structures have also been shown theoretically to confer robustness onto regulatory pathways [11,12].

Various artificial perturbations of the cell, such as comprehensive gene knockout or knockdown experiments [13,14], have been useful tools in studying robustness. Such experiments gave rise to the surprising observation that many knockouts/knockdowns have no detectable phenotype under the conditions tested, and several types of robustness hypotheses, including buffering by duplicated genes, have been proposed to explain this pattern [2,15]. A complementary type of perturbation for which robustness has been less explored is increases in gene copy number. Here we focus on a specific type of increase: the introduction of one or more extra chromosomes, (*e.g.*, aneuploidy). Our question is to what degree the cellular systems for modulating gene expression noise also compensate for such radical changes in gene dosage. Our central hypothesis is that natural selection will have shaped the degree of transcriptional robustness for a gene in part based on whether any epistastic interactions involving that gene interfere with cellular function. Epistasis is the phenomenon whereby the phenotypic effect of variation in one gene depends on which variants of other genes are present [16]. One example is the negative epistasis seen between duplicate genes: Dean and colleagues [17] have shown that knocking out pairs of duplicate genes is more detrimental than expected based on their individual knockout effects and the overall distribution of double knockout effects among pairs of single copy genes. The term epistasis is employed in two senses [18,19]. Statistical epistasis implies that models of non-interacting genes or loci fail to predict the effects of multi-gene variation. However, in this work, we are concerned instead with biological epistasis, which involves a mechanistic, molecular association between two genes. In a strict sense, biological epistasis requires the comparison of variation (natural or artificial) at two genetic loci and the demonstration that a phenotype can only be predicted with knowledge of the allele present at both loci [16]. Here, our interest is more general and focuses instead on the types of molecular architecture that can give rise to such genetic observations.

Studies of gene expression in aneuploids have led to several hypotheses as to the patterns of expression change in these cells [20]. Generally, there is expected to be a global dosage effect yielding a relative increase in expression for genes on the extra chromosome(s) [21,22]. However, this effect will be modulated by dosage compensation resulting from the transcriptional regulatory effects discussed above [23,24]. Indeed, the detrimental effects of aneuploidy parallel those of gene expression noise, and mechanisms such negative autoregulation should allow cells respond to both types of insult.

Here, we used the response of *Saccharomyces cerevisiae *(bakers' yeast) cells to aneuploidy [21] to test our hypothesis that genes with more protein-protein interactions will show stronger buffering against changes in dosage. This hypothesis is related to the *dosage balance hypothesis *[25-27], that argues that natural selection will tend to disfavor individual changes in dosage for genes that are found in central parts of biological networks (such as transcription factors and kinases) due to the imbalance in network stoichiometry that results [28]. Such dosage constraints can be overcome by the duplication of the entire genome, which, to an initial approximation, leaves relative gene dosage unaltered. We argue that highly epistatic genes, which the dosage balance hypothesis predicts to be under selection against changes in copy number, will also tend to possess transcriptional regulatory mechanisms that provide robustness against expression noise.

Our analyses are based on the pioneering study of Torres *et al. *[21], who created aneuploid strains of *S. cerevisiae *for 13 of the 16 chromosomes (although some of these strains also carry extra copies of one or two other chromosomes as well). These researchers found that aneuploid strains tend to be slower growing than wild-type cells and that there was an apparent general trend of over-expression of genes on the aneuploid chromosome (but see [20] ). Here, we employ these gene expression data to ascertain the response of non-aneuploid genes to the doubling of dosage of aneuploid genes. We hypothesized that the gene expression response of genes having a protein-protein interaction with an aneuploid chromosome-encoded protein would be different than that for genes lacking such interactions.

## Results

### Protein-protein interactions and aneuploidy

In their otherwise haploid *S. cerevisiae *lines, Torres *et al. *[21] used microarrays to measure the changes in gene expression resulting from aneuploidy. Taking the normalized expression ratios (as compared to non-aneuploid controls, see *Methods*) from these experiments, we examined the degree of aneuploidy-induced expression variation for the genes *not *on the aneuploid chromosome. We note parenthetically that this approach avoids the issue of how to normalize the measured expression levels of the genes on the aneuploid chromosome relative to those measured from the remainder of the genome [20].

**Figure 1:**
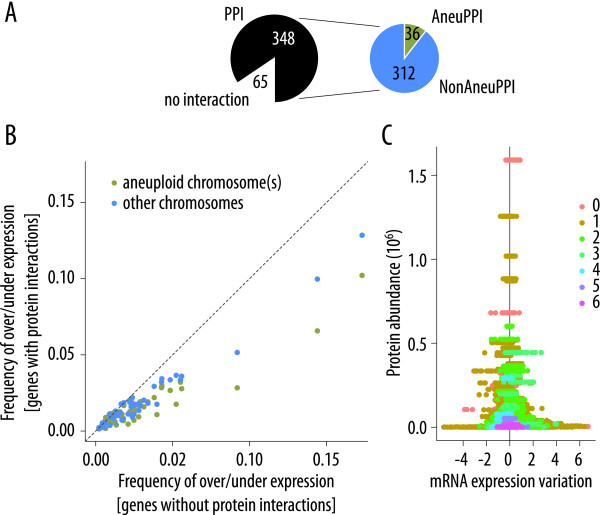
**Correlation between protein-protein interactions and mRNA expression variation**. (**A**) Definition of the three interaction datasets considered. PPI consists of all non-aneuploid chromosome genes coding for a protein with at least one interaction (348 genes out of 413 in the example considered here). Any genes with at least one protein interaction with a protein encoded on the aneuploid chromosome are in AneuPPI (36 here); all other members of PPI are placed in NonAneuPPI (312). (**B**) Plot of the frequency of the genes with increased or decreased mRNA expression (*y-*axis; 1.3-fold change, see *Methods*) coding for a protein interaction with a protein encoded either on an aneuploid chromosome, AneuPPI (green dots), or on a different chromosome, NonAneuPPI (blue dots), against the proportion of genes without interactions that showed changed mRNA expression for each aneuploidy microarray experiment (*x*-axis). (**C**) Scatter plot of protein abundance (protein molecules per cell) versus mRNA expression variation. High abundance proteins show low mRNA variation (Spearman rank correlation; *P *< 10^-15^). The number of protein-protein interactions is color-coded as illustrated by the legend at right. Proteins with a high degree of interaction are of both low abundance and low mRNA expression variation (Spearman rank correlations, *P *< 10^-15^).

We first determined the set of genes from each aneuploid line that showed either an increase or decrease in expression (*i.e.*, had greater than ± 1.3-fold variation compared to the control strain, *Methods*). We next defined the set of genes that have at least one protein-protein interaction (hereafter PPI, Figure 1A). The genes in PPI are less likely to show post-aneuploidy expression variation than are non-interacting genes (Figure 1B; Wilcoxon unilateral signed-rank test, *P *< 10^-9^). Moreover, those genes coding for a protein possessing an interaction with a protein encoded on the aneuploid chromosome (hereafter AneuPPI, Figure 1A) showed less expression variation than do genes with interactions to proteins encoded on non-aneuploid chromosomes (hereafter NonAneuPPI; Figure 1A &1B; Wilcoxon unilateral signed-rank test, *P *≈ 0.0002). We questioned whether these differences between AneuPPI and NonAneuPPI could be due to differences in the average number of protein-protein interactions per gene for the two sets. However, we found that the average number of interactions was very similar: 1.493, and 1.495 for NonAneuPPI and AneuPPI respectively. These distributions are not significantly different. (Kolmogorov-Smirnov test, two sided, *P *> 0.5). So far, we have lumped over- and under-expression together in these analyses. When we split the data by considering the direction of variation (and in so doing reduce the power of the tests), we again find that genes from the set PPI are under-represented among both the genes with an increase and with a decrease in expression (Wilcoxon unilateral signed-rank test, *P *< 10^-4^). Likewise, the AneuPPI set showed fewer under-expressed genes relative to NonAneuPPI than expected (Wilcoxon unilateral signed-rank test, *P *< 10^-5^). No significant difference in the proportion of over-expressed genes was seen between AneuPPI and NonAneuPPI (Wilcoxon unilateral signed-rank test, *P *≈ 0.13).

We next asked whether any of our genes sets showed a difference between the proportion of over-expressed and under-expressed genes. For the dataset as a whole, as well as PPI and NonAneuPPI, there was no such difference (*i.e.*, the expression variation distributions appear symmetric, Wilcoxon unilateral signed-rank tests, *P *> 0.05). However, for the AneuPPI set, there were significantly more over-expressed genes than under-expressed ones (Wilcoxon unilateral signed-rank tests, *P *≈ 0.002), consistent with the expectation that dosage compensation mechanisms were at work to increase their expression to a level closer to that of their aneuploid partners.

Gene expression noise is highest in lowly expressed genes [29], and it is possible that the signals in Figure 1B might be due to co-variation of protein-interaction degree and expression level. We thus compared protein abundance [an expression proxy taken from Ghaemmaghami et al., [30]] to mRNA expression variation. As expected, high abundance proteins show low expression variation (Spearman rank correlation test, *P *< 10^-15^). However, proteins with high protein interaction degree are of both of low abundance and low mRNA expression variation, refuting the hypothesis of a simple co-variation with expression level (Spearman rank correlation tests, *P *< 10^-15^; Figure 1C). It is also worth noting the relatively limited range of protein interaction degree inherent in this figure, a feature which makes it difficult to measure any association between interaction degree and expression variation.

**Figure 2:**
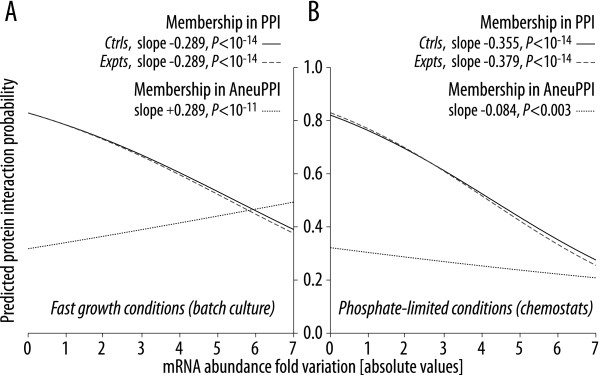
**Expression variability is increased for genes interacting with the aneuploid chromosome under conditions of rapid cell division**. The variability of mRNA expression level (absolute value of fold change) was used as a predictor of the presence of a protein interaction using logistic regression. Three models were tested. The first two predict protein interaction presence (*i.e.*, membership in the set PPI) using the basal mRNA expression variation from either the control (*Ctrls*, no aneuploid chromosome; solid lines) or the experimental (*Expts*, presence of an aneuploid chromosome; dashed lines) strains. The third uses expression data to predict the presence of an interaction with a protein encoded on an aneuploid chromosome (*i.e.*, membership in AneuPPI; dotted lines), given that the gene in question is already a member of PPI. (**A**) Expression measured under rapid growth conditions (batch culture). (**B**) Expression measured under slow growth conditions (chemostat, phosphate-limited).

The above analyses rest on the introduction of a somewhat arbitrary cutoff for defining genes with post-aneuploidy expression variation. To assess whether this choice might have biased our conclusions, we employed a logistic regression model to evaluate whether measured mRNA variation was predictive of the existence of protein-protein interactions involving a given gene's product. For both the rapid growth batch cultures and the phosphate-limited chemostat ones, mRNA expression variation is an excellent predictor of the presence of an interaction (Figure 2A &2B, respectively). Specifically, we tested three models. For the first two models, we evaluated whether the level of mRNA expression variation predicted the presence of a protein interaction both for the control experiments of Torres *et al. *[21], where no aneuploid chromosomes were present, and for the aneuploid strains themselves. In both cases, there was a strong negative association between high expression variation and the presence of a protein interaction (*P *< 10^-14 ^in all cases, Figure 2). Interestingly, this association was reversed when we considered genes with an interaction involving the aneuploid chromosome under fast growing conditions. In that case, we found that mRNA expression variation was positively associated with the presence of such an aneuploid protein interaction (*P *< 10^-11^, Figure 2A). We interpret this result as evidence of some regulatory mechanism that tries to balance the expression of genes off the aneuploid chromosome with their interaction partners that are on this chromosome. However it is important to note that, under phosphate-limiting conditions (slow growth), the pattern among the aneuploid interactions was similar to the negative association seen when all interactions were considered (*P *< 0.003, Figure 2B). We note that our results are robust to the removal of outliers in gene expression variation (points with greater than three-fold variation; data not shown).

### Transcriptional factor interactions mediate the expression response to aneuploidy

If indeed there were a tendency for tighter regulation of epistatic genes in response to noise, one would expect that control to be evident in the transcriptional regulatory network. We thus examined the aneuploidy-induced changes in expression among transcriptional factors (TFs) and their target genes. Changes in expression post-aneuploidy are more common among TFs than in genes in the genome at large (Fisher's Exact Test, *P *< 10^-5^). In phosphate-limited conditions (slow growth), genes that are the target of aneuploid chromosome-encoded TFs are more likely to be over-expressed than genes in the genome at large (Fisher's Exact Test, *P *≈ 0.01). Simultaneously, genes from this same set are also more likely to be under-expressed compared to the rest of the genome (Fisher's Exact Test, *P *≈ 0.001). Strikingly, this pattern is reversed in the rapidly growing batch cultures, where the enrichment of over-expressed genes is greater than that of under-expressed genes (*P *< 10^-10 ^and *P *< 10^-5^, respectively). Presumably, these results derive from the logic of the transcriptional regulatory network and its growth condition-dependant patterns of transcriptional activators and repressors. Unfortunately, we currently lack the genome-scale data to better explore how the logic of this network gives rise to different expression patterns depending on conditions.

## Discussion

Many authors have explored the close connection between epistasis and robustness. In both digital and some experimental organisms, the phenomenon of synergistic epistasis (double mutants showing smaller phenotypic effects than the respective single mutants would predict) bears a strong resemblance to mutational robustness [31,32]. There is also debate on whether such robustness/epistasis represents an adaptation or is simply the consequence of the general structure of genetic networks [2,33]. A similar arena for the observation of epistasis is in changes in gene dosage or expression. The implication of the dosage balance hypothesis is that such changes may have fitness costs if a given gene's partners do not undergo similar dosage changes. Here we have hypothesized that the potential costs of dosage imbalance have led to the evolution of mechanisms for gene expression robustness that respond to expression changes in one gene with compensating changes in its partners [34]. Because we cannot direct assess a gene's potential to suffer from dosage imbalance, we used the possession of a protein-protein interaction as (imperfect) marker for such genes. It is clear that such long distance genetic associations are seen in the aneuploidy lines: the aneuploidy "allele" interacts with numerous wild-type alleles on the remaining chromosomes and alters their phenotype (expression). Moreover, as our hypothesis suggests, we do find a trend of greater expression compensation among interacting genes. Thus, gene products interacting with proteins encoded on the aneuploid chromosome are more likely to increase in expression than to decrease, just as one would expect if their expression level were responding to the increased dosages of their partners. Notably this association is not seen in the transcriptional regulatory network: instead of the decreased expression variability seen with protein-protein interactions, targets of aneuploid regulators show the expected pattern of greater than average change in expression. These patterns do not demonstrate epistasis in the classic sense, since variation in the wild-type, non-aneuploid, genes has not been examined [16]. However, it is obvious that the potential exists, since changes in upstream regulatory regions of these genes might abolish the observed post-aneuploid expression changes. More importantly, the associations we observe are precisely the types of genetic architectures that we would expect to give rise to the experimental observation of epistasis.

At first blush, the association of protein-protein interactions and reduced expression variation would seem to be in contrast to the dosage balance hypothesis [25-27]. We prefer to see the existence of such buffering as evidence of the importance of maintaining proper dosage balance in the cell. In this view, the potential deleterious effects of changes in dosage have been partly mitigated by the evolution of mechanisms for noise reduction and coordinated regulation. We also note that the increase in glucose uptake among aneuploid cells observed by Torres *et al*. [21], is in keeping with our previous argument that selection for increased gene dosage of glycolytic genes was one force that contributed to the preservation of a similar large scale duplication in the ancestor of bakers' yeast, in that case a whole-genome duplication [35].

Both epistasis [17,36] and noise [37] are ubiquitous in cells. Thus, the existence of mechanisms to compensate for expression noise in complex cellular networks is not surprising. In fact, a number of the mechanisms responsible for this robustness are known, including the structure of metabolic reactions [38,39], as well as features of the transcriptional regulation of single genes [4,40,41] and of transcriptional regulatory networks [5,11,12,42]. An additional mechanism, not considered here, is regulation at the level of protein degradation. Intriguingly, Torres and coauthors found that their aneuploid strains showed evidence of increased protein degradation rates for proteins encoded on the aneuploid chromosomes [21]. The fact that aneuploidy is both rare and detrimental [21] means selection for aneuploid robustness itself is very unlikely. Instead, the reaction of yeast cells to aneuploidy opens a window into the general cellular response to expression changes. Aneuploidy is also an interesting test of expression buffering because one can imagine at least two general strategies for such buffering. In a gene-by-gene model, the expression of each gene is tightly but individually regulated, with the overall expression levels tuned among the epistatic genes by natural selection. Such a system implicitly assumes unchanging copy-numbers among the genetic modules. However, our results suggest that, at least in some cases, a different mechanism is at work, because such a gene-by-gene model cannot explain the over-abundance of up-regulated genes with interactions to the aneuploid chromosome. Instead, such results suggest that at least some modules have the ability to sense the expression levels of certain member genes and adjust the expression of their partners appropriately. Such a system has obvious advantages, given that while DNA is unable to sense distant changes in expression or copy-number, mRNA molecules can do so.

## Conclusion

Our analysis shows only association, not causation. Nonetheless, an association between molecular interactions and regulatory modules obeys an certain evolutionary logic in avoiding potentially damaging mismatches in abundance between proteins that work together. It will be difficult but rewarding to tease apart the various transcriptional modules to see the frequency with which genes are expressed in proportion to the abundance of their interaction partners. Likewise, it is also unclear exactly which types of environmental and cellular insults cells have evolved to be robust against [2], and it will be interesting to untangle this problem

## Methods

### Data collection and pre-processing

We used mRNA expression variation determined from an extensive set of aneuploid chromosome lines of otherwise haploid *S. cerevisiae *from Torres *et al. *[21]. These data include 37 microarray experiments in batch culture (fast growth conditions), 14 experiments in chemostat under phosphate-limiting conditions (steady-state/slow growth conditions), and their respective controls. For each such experiment (comprising an aneuploid strain compared to a wild-type one in the same conditions), we first excluded all genes on the aneuploid chromosome from the dataset. We then obtained the ratio of expression between the aneuploid and wild-type strains for each gene. Cases where the aneuploid was 1.3 fold higher or lower in expression were defined as variable under aneuploidy.

We gathered protein-protein interaction data on 4,988 *S. cerevisiae *proteins from the Database of Interacting Proteins (DIP; [43]), comprising 24,864 total pairwise interactions. Using these interactions we defined three sets of genes. In the set PPI are all genes not on the aneuploid chromosome (see above) that have a protein-protein interaction based on the DIP data. PPI has two subsets (Figure 1A). AneuPPI consists of members of PPI with at least one interaction partner encoded on the aneuploid chromosome. All other members of PPI are placed in NonAneuPPI.

To control for the effect of expression/translation level on our comparisons we used protein abundance values for 3,868 proteins taken from Ghaemmaghami *et al. *[30]. Our analysis of transcriptional regulation and aneuploidy was based on a list of 203 transcriptional regulators and their targets from Harbison *et al. *[44]. To identify such targets we used the default *P*-value threshold of 10^-3 ^described by these authors.

### Binomial Logistic Regression

A logistic regression model [45] was used to evaluate the relationship between gene expression change after aneuploidy and the presence of a protein-protein interaction. We used the absolute value of the expression variation data described above (before the application of the 1.3-fold cutoff) as our predictor. We asked whether such expression change was predictive of the presence of two of the types of protein-protein interactions in Figure 1A (PPI and AneuPPI, dashed and dotted lines in Figure 2, respectively) as well as the presence of a protein interaction in Torres *et al.*'s [21] control experiments (solid lines in Figure 2). Conceptually, we are asking whether a given gene, drawn at random from the nonaneuploid genes, possesses a protein interaction, given its expression change after aneuploidy. We use a likelihood-ratio test to ask whether adding expression information (allowing the slope in Figure 2 to be nonzero) significantly improves our ability to make such a prediction as opposed to simply using the overall frequency of protein-protein interactions as our predictor (having the slope constrained to zero; chi-square distribution with one degree of freedom). The analysis was implemented in the package mlogit v0.18 in R [46].

## List of abbreviations used

TF: Transcriptional Factor; DIP: Database of Interaction Proteins; PPI: set of genes that have at least one protein-protein interaction; AneuPPI: set genes coding for a protein possessing an interaction with a protein encoded on the aneuploid chromosome; NonAneuPPI: set of genes with interactions to proteins encoded on non-aneuploid chromosomes.

## Authors' contributions

GCC and MB designed the study. MB performed the computational and statistical analyses. Both authors read and approved the final manuscript.
